# From Deep‐Fat Frying to G‐Frying: Exploring Absorption Dynamics of Oil Fractions in Fried Foods

**DOI:** 10.1002/fsn3.72391

**Published:** 2026-09-20

**Authors:** Arash Ghaitaranpour, Mohsen Esmaiili, Zoleykha Fahimi vajargahi, Michael O. Ngadi

**Affiliations:** ^1^ Department of Food Science and Technology Urmia University Urmia Iran; ^2^ Department of Bioresource Engineering McGill University Montreal Canada

**Keywords:** deep‐fat frying, novel frying methods, oil absorption behavior, oil fractions

## Abstract

There are some valuable reviews about strategies to reduce oil content; however, they do not thoroughly illustrate oil absorption patterns across different frying methods. The present study provides a comprehensive comparison of various frying methods, including deep‐fat frying, hot‐air frying, microwave frying, G‐frying, pressure frying, vacuum frying, and infrared frying, focusing on the unique oil absorption patterns associated with each method. Moreover, the current review explains underlying mechanisms responsible for oil absorption during different frying phases, such as absorption behavior of each fraction of total oil content. This detailed analysis is essential for understanding how alternative frying technologies affect oil absorption differently from traditional methods. In deep‐fat frying, oil absorption mainly occurs during the cooling phase, whereas pressure frying, vacuum frying, hot‐air frying, microwave frying, G‐frying, and infrared frying each exhibit distinct oil absorption patterns due to their different processing conditions. Differences in factors like the amount of oil surrounding the product and heat transfer mechanism lead to different oil penetration mechanism in emerging frying technologies. By highlighting the differences in oil absorption patterns and mechanisms, the present review provides practical insights for both researchers and food processing industries. This can guide future research toward developing more efficient frying technologies that reduce oil content while maintaining desirable food qualities, thus meeting the increasing demand for healthier fried foods.

## Graphical abstract

This review provides a comparative analysis of oil absorption across conventional and emerging frying technologies, including hot‐air, microwave, G‐frying, pressure, vacuum, and infrared frying. It examines the migration of surface oil, structural oil, and penetrated surface oil during frying and cooling, highlighting technology‐specific absorption patterns and mechanisms. This framework provides new insights into controlling oil uptake while maintaining the desirable quality of fried foods.

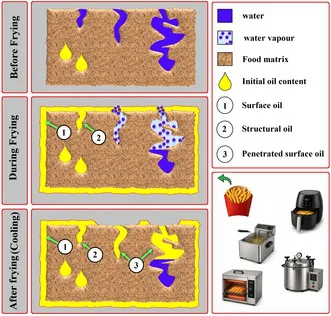

## 1 Introduction

Deep‐fat frying consists of simultaneous heat and mass transfer, in which cooking in oil/fat gives desirable quality characteristics such as taste, texture, appearance, and odor to food products (Guillermo et al. 2021). In general, frying has a higher heat transfer rate compared to other cooking methods, and the high temperature causes a sharp reduction in the food crust moisture content and increases chemical reactions of various food components, including protein denaturation and carbohydrate caramelization (Pankaj and Keener 2017). The high temperature of frying develops a favorable texture in the product where a dry and crispy crust is usually formed around the moist and soft core (Su, Li, et al. 2024; Voong et al. 2018). As described in Table 1, in addition to inducing physicochemical changes in food, the high temperatures used during frying also cause significant changes in the frying oil itself (Valle et al. 2024). Extensive research has investigated the effects of thermal treatment on edible vegetable oils, demonstrating that prolonged exposure to high temperatures accelerates oxidation, hydrolysis, polymerization, and the formation of degradation products, thereby altering the physicochemical properties, oxidative stability, and nutritional quality of the oil (Giuffrè et al. 2018; Giuffrè et al. 2017; Valle et al. 2024; Wang et al. 2026). These thermal changes not only influence oil quality and frying performance but may also affect the interaction between oil and food during frying.

**TABLE 1:** Effects of different frying methods on food properties and oil chemical composition.

Frying method	Operating conditions	Impact on food properties	Impact on oil chemical composition	Main advantages	Main limitations
Deep‐fat frying	160°C–190°C, atmospheric pressure	Rapid moisture loss, crust formation, desirable color and flavor, high oil uptake, texture development	Accelerates oxidation, hydrolysis, polymerization, formation of polar compounds and free fatty acids	Excellent sensory quality, simple process	High oil uptake, oil degradation, lower nutritional quality
Vacuum frying	80°C–140°C, reduced pressure	Lower moisture loss rate, reduced oil uptake, better color retention, improved retention of heat‐sensitive nutrients	Slower oxidation and thermal degradation due to reduced oxygen and lower temperature	Better nutritional quality, extended oil life	Higher equipment cost, longer processing time.
Air frying	160°C–200°C, circulating hot air with minimal oil	Crisp surface, lower oil absorption, different texture compared with deep frying	Minimal oil degradation because only a small amount of oil is used	Low‐fat product, healthier profile	Produce less desirable flavor and texture
Pressure frying	160°C–180°C under elevated pressure	Faster Frying, greater moisture retention, tender interior, moderate oil uptake	Oil degradation similar to deep frying but with shorter frying time	Improved juiciness and frying efficiency	Specialized equipment required
Microwave frying	Microwave heating combined with oil	Rapid internal heating, shorter frying time, reduced oil uptake, improved moisture distribution	Reduced thermal exposure may decrease oxidation; localized overheating may occur	Faster process, lower energy consumption	Uneven heating if poorly controlled
Infrared frying	Infrared radiation with or without oil	Rapid surface heating, enhanced crust formation, lower oil uptake than conventional frying	Shorter heating time may reduce oxidation and polymerization	High heating efficiency	Limited industrial application
Hybrid frying	Combination of two or more technologies	Improved texture, lower oil absorption, better nutrient retention, more uniform cooking	Lower oil deterioration due to shorter frying time and/or reduced temperature	Combines advantages of different frying methods	Process optimization is more complex
G‐frying	160°C–190°C	Reduced oil uptake, improved texture and product uniformity (reported in limited studies)	Preliminary studies indicate reduced oil oxidation due to shorter heating exposure	Potential for healthier fried products	Limited research and industrial validation

The unique organoleptic properties of fried products, including taste, color, texture, and odor, make them popular among consumers (Zhang et al. 2020). The wide range of fried foods and their ubiquity in fast‐food restaurants have raised their overall consumption globally. According to a survey conducted a few years ago, an increase of 208 billion dollars had been estimated for food sales in the supermarkets, restaurants, fast‐food restaurants, and other food retail centers of the United States by 2020 (Pankaj and Keener 2017).

**FIGURE 1:**
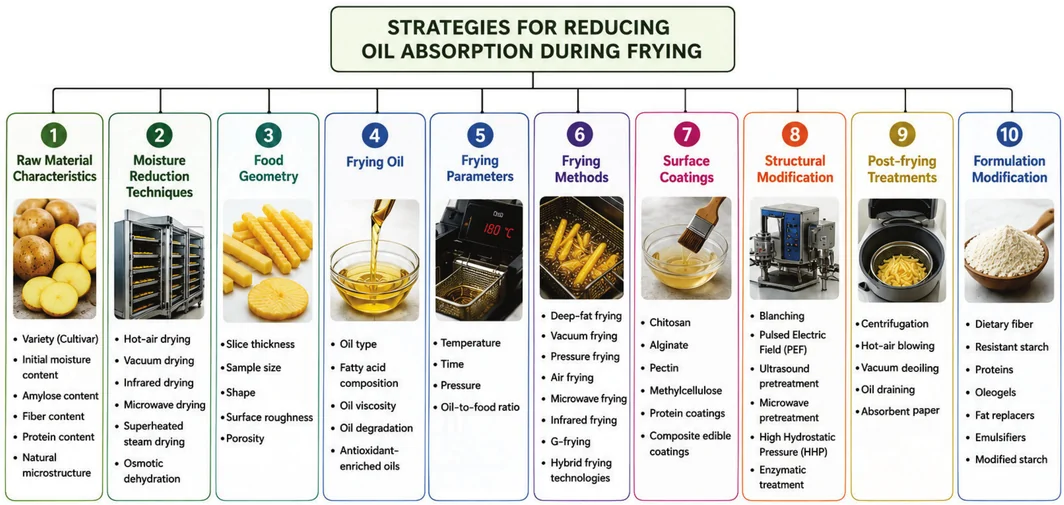
Graphical overview of the main strategies for reducing oil absorption during frying. HHP, High hydrostatic pressure; PEF, Pulsed electric field.

On the other hand, fried food absorbs a lot of oil during traditional frying, and the amount of oil absorption can reach up to 50% of the food total weight (Xie et al. 2022). Since excessive consumption of fried foods with high oil and calorie contents elevates the risk of obesity, high blood pressure, cardiovascular diseases, diabetes, and cancers (Valle et al. 2024; Zhang et al. 2016), consequently, reducing oil absorption in fried foods is one of the most important areas of research in food science (Valle et al. 2024). Various approaches have been proposed to achieve this goal, as summarized in Figure 1. The growing population of consumers who pay more attention to healthy fried foods with lower TOC has given rise to the demand for the development of new alternative frying technologies. Even food processing industries are seeking novel frying methods that result in the same properties of the fried food as conventional frying while having less oil absorption (Zhang et al. 2020). Alternative frying technologies such as vacuum, hot‐air, and microwave frying, or their combinations, have been applied to decrease oil absorption (Liberty et al. 2019). Nevertheless, reducing oil content without compromising the characteristic sensory attributes of fried foods remains challenging because oil contributes not only to caloric value but also to flavor development, mouthfeel, and texture formation.

Recent review papers have comprehensively discussed the mechanisms of oil absorption in fried foods, the effects of frying conditions, and strategies for reducing total oil uptake (Zhang et al. 2025). Other reviews have focused on emerging frying technologies, including vacuum frying, microwave‐assisted frying, ultrasound‐assisted frying, pulsed electric field applications, and edible coatings as approaches to reduce oil content in fried products (Huang et al. 2025; Manoharan et al. 2024). Although these reviews provide valuable insights into total oil uptake and frying process optimization, they largely consider absorbed oil as a single entity and do not specifically address the selective absorption behavior of different oil fractions. Unlike previous reviews that primarily evaluate total oil content (TOC) as a single indicator of frying performance, this review emphasizes that the total oil content consists of surface oil (SO), penetrated surface oil (PSO), and structural oil (STO), as described by other researchers (Bouchon et al. 2003).

Based on the above literature, frying‐induced changes in foods are influenced by several factors, including frying technique, oil type, process temperature, and frying time. Although numerous studies and recent reviews have investigated oil absorption mechanisms and strategies for reducing total oil uptake (Huang et al. 2026; Zhang et al. 2026), the relationship between frying technology and fraction‐specific oil migration remains insufficiently understood. In particular, limited attention has been given to whether different technologies reduce oil uptake by preventing oil penetration, limiting surface oil retention, or modifying post‐frying absorption during cooling. Therefore, the present review compares conventional and emerging frying technologies from the perspective of the three major oil fractions (SO, PSO, and STO) rather than total oil content alone. By integrating mechanistic evidence from different frying systems, this review aims to clarify how each technology influences specific stages of oil migration, identify inconsistencies and knowledge gaps in the current literature, and provide insights for designing frying processes that minimize undesirable oil uptake while maintaining product quality.

## 2 Materials and Methods

**FIGURE 2:**
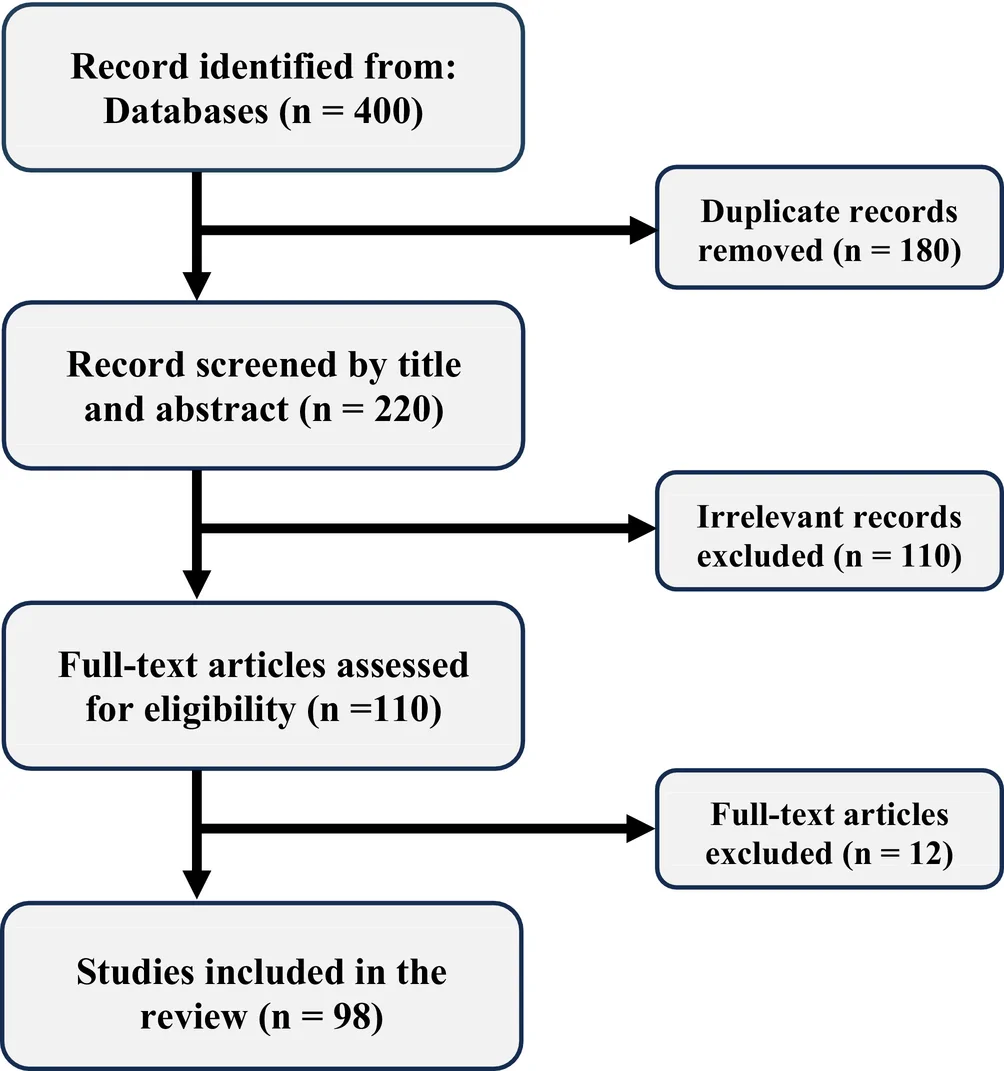
Literature search and study selection process for the comprehensive review on absorption dynamics of oil fractions in fried foods.

As described in Figure 2, the literature search was conducted using Scopus, PubMed, ScienceDirect, Google Scholar, Wiley Online Library, and SpringerLink to identify studies published between 2000 and 2026. The search included terms such as “deep‐fat frying,” “vacuum frying,” “air frying,” “microwave frying,” “pressure frying,” “infrared frying,” “G‐frying,” “oil absorption,” “oil uptake,” “oil fraction,” and “oil absorption modeling.” Only English‐language journal articles and book chapters were considered. Studies investigating oil absorption mechanisms, conventional and emerging frying technologies, and experimental oil uptake were included, whereas duplicate records, conference abstracts, non‐English publications, studies without full text, and articles unrelated to oil absorption were excluded.

## 3 Oil Distribution in Fried Food

**FIGURE 3:**
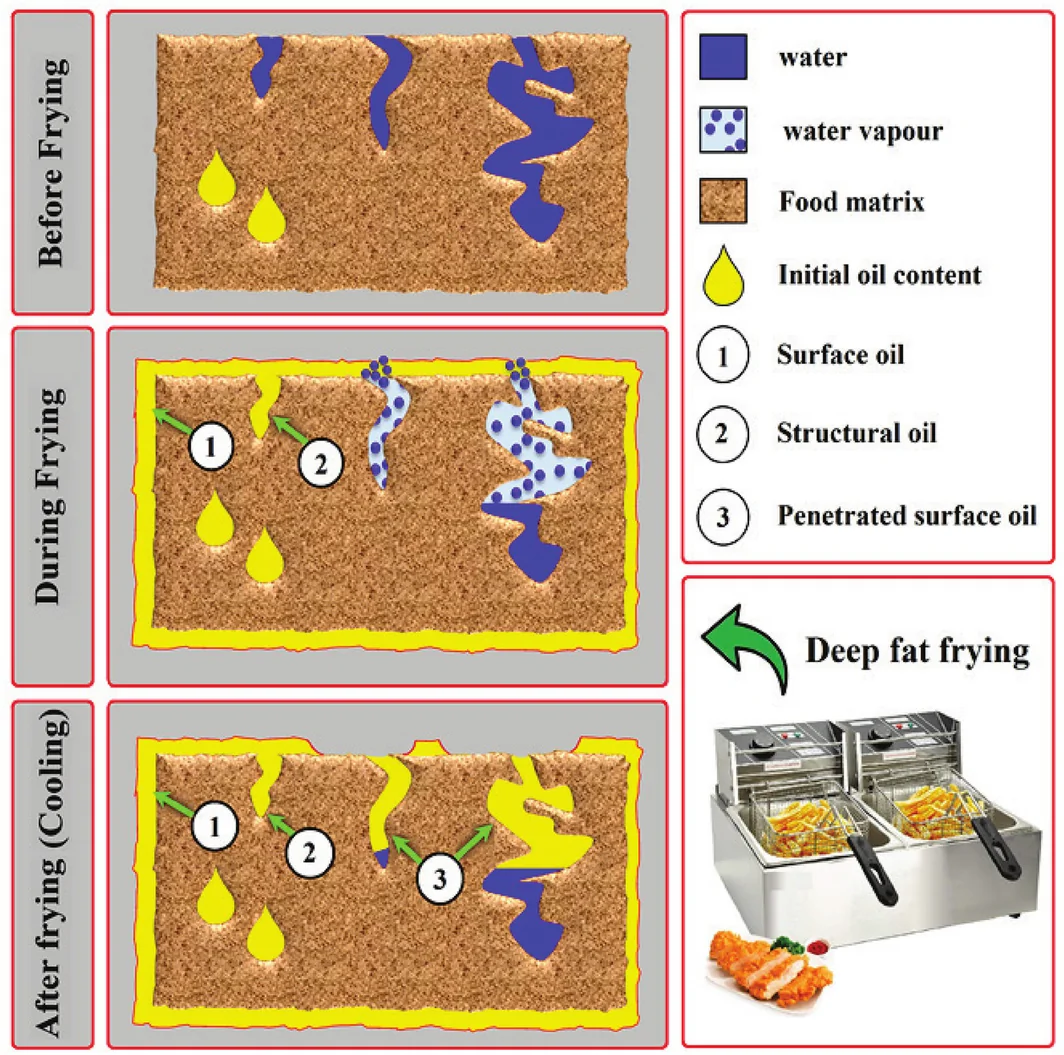
Oil and water distribution in a food material before, during and after deep fat frying.

Oil has dramatic effects on the organoleptic and nutritional properties as well as the calorie content of fried foods (Eichenlaub and Koh 2015). Total oil content (TOC) in fried foods is a critical factor that causes health issues. During frying, two countercurrent mass transfer processes occur. The first is water transfer, which happens in two stages. Initially, water and water‐soluble components from the inner parts of the food move to the surface of the crust. In the next stage, the water evaporates from the crust. The second process is oil absorption, where oil from the frying medium penetrates the food, moving in the opposite direction of the water. To reduce TOC in fried foods without compromising quality, it is essential to understand the different categories of absorbed oil. Previously, it was believed that most oil absorption occurred during frying due to moisture evaporation through the food's capillary tubes (Zhang et al. 2020). However, recent studies have shown that the final TOC of a fried food depends not only on the frying process but also on the post‐frying conditions (Eichenlaub and Koh 2015). In fact, the major amount of oil is absorbed during cooling which causes water vapor to condense in the food pores and creates negative pressure, so the oil is sucked from the surface into the pores (Zhang et al. 2020). Researchers have divided the TOC of fried foods into three different fractions, each having its own specific absorption mechanism (Bouchon et al. 2003; Pedreschi et al. 2008):

- Structural oil (STO), which represents the penetrated oil absorbed during frying (Figure 3) (Ghaitaranpour et al. 2021; Lumanlan et al. 2020; Zhang et al. 2018).
- Surface oil (SO) which remains on the food surface and does not penetrate into its microstructure (Figure 3).
- Penetrated surface oil (PSO), which indicates the oil sucked into the food after frying and during cooling (Figure 3).

Researchers divided the TOC of French fries into three fractions: (a) STO, (b) PSO, and (c) SO (Bouchon et al. 2003). They stated that a small amount of oil penetrated into the product during frying, revealing that oil absorption and water loss are not always simultaneous phenomena. Furthermore, they mentioned that after cooling, the oil either remained on the surface of the fried product or was sucked into the microstructure of the porous crust (Ghaitaranpour et al. 2021; Pedreschi and Enrione 2014). As shown in Table 2, the TOC of fried food is composed of PSO, STO, and SO, respectively (Lumanlan et al. 2020; Pedreschi et al. 2008), and the unique condition of each frying method can alter them in a specific way. Considering the fact that the mentioned oil factions have different absorption patterns, in this review, the variations in each fraction have been investigated considering these three general categories.

**TABLE 2:** Effect of frying method condition on absorption of different oil fractions.

Oil fraction	Frying condition	Summary and mechanism of action	References
Structural oil (STO)	Surrounding oil during frying	A lack of sufficient surrounding oil during hot‐air frying results in a decrease in STO.	Abd Rahman et al. (2017), Ghaitaranpour et al. (2021), Wang et al. (2021)
	Surrounding pressure during frying	High pressure during frying increases the water boiling temperature, subsequently decreasing STO.	Pankaj and Keener (2017), Rosida et al. (2018), Zhang et al. (2020)
		Low pressure increases evaporation rate and, in some cases, can decrease STO.	Ayustaningwarno et al. (2020), Correa et al. (2017), Motamedzadegan et al. (2016), Soto et al. (2021), Wexler et al. (2016).
	G‐force during frying	G‐force reduces porosity, which in turn decreases STO.	Khalilian et al. (2021)
	Post frying condition	Higher vacuum breaking rate decreases STO.	Mir‐Bel et al. (2009), Soto‐Jover et al. (2016)
	Heating mechanism	IR‐Frying leads to higher water evaporation, decreasing STO.	Devi et al. (2021), Rahimi et al. (2018)
Surface oil (SO)	Surround oil during frying	Insufficient surrounding oil during frying leads to a decrease in SO.	Ghaitaranpour et al. (2021)
	Changing food surface properties during frying	IR heating creates a smoother crust layer more quickly, leading to a decrease in surface oil absorption.	Devi et al. (2021), Rahimi et al. (2018)
	Post frying condition	G‐force removes SO from the crust.	Khalilian et al. (2021)
		Decreasing pressure after frying reduces SO by rising evaporation.	AlMohaimeed (2017), Dehghannya and Ngadi (2021), Mir‐Bel et al. (2009), Soto‐Jover et al. (2016)
Penetrated surface oil (PSO)	Surround oil after frying	G‐force reduces SO after frying, preventing the rise in PSO during cooling.	AlMohaimeed (2017), Dehghannya and Ngadi (2021), Mir‐Bel et al. (2009), Soto‐Jover et al. (2016)
		Insufficient surrounding oil after hot‐air frying prevents PSO increase.	Abd Rahman et al. (2017), Ghaitaranpour et al. (2021), Wang et al. (2021)

## 4 Important Mechanisms of Oil Absorption

The TOC is one of the most essential quality indicators of fried foods, as it is related to consumer's health (Bouchon et al. 2003; Pedreschi et al. 2008). With the continuous development of societies, public knowledge about healthy diets has expanded, and the importance of low‐fat and low‐calorie diets has risen. Moreover, from the perspective of food processing industries, lower TOC of food products can lead to lower production costs and increase the profitability of related factories (Xie et al. 2022). Understand the mechanism of oil absorption is one of the first steps in reducing TOC, as it can help optimize the performance of the frying equipment and produce higher‐quality products. In addition, a better understanding of oil absorption action can help develop more efficient methods of oil reduction (Liberty et al. 2019). As mentioned earlier, scientists have divided the TOC of fried food into three main groups (SO, PSO, and STO), each being absorbed into the food based on its own specific way (Bouchon et al. 2003; Pedreschi et al. 2008).

### 4.1 Mechanism of SO Absorption (Adhesion and Drainage)

Absorption of this oil fraction is a surface phenomenon, and its content depends on the balance between adhesion and drainage after the product is removed from the frying medium (Arslan et al. 2018; Bouchon et al. 2003; Pedreschi et al. 2008). Adhesion, which is a significant factor in oil absorption, is the ability of oil to adhere to the external surface of the product. It has been claimed that degraded oil has more tendency to adhere to the food surface due to the increase in the oil viscosity caused by the oil degradation (Bouchon et al. 2003; Lumanlan et al. 2020). Scientists have attributed this increase to the formation of polymer products, resulting in a decrease in the quality of the frying oil. It has also been declared that the increased oil viscosity and the surface properties of the fried product are the vital factors governing the oil absorption after the product is removed from the frying medium (Arslan et al. 2018). On the other hand, drainage is the removal of SO as a result of gravitational forces. Theoretically, when a solid is removed from a liquid, a thin layer of the liquid covers it. Researchers have demonstrated that the gradual removal of the film formed on fiber coating from pure adhesive liquids such as oils can be expressed by Landau's law. At high removal rates, the reduction in the formed film thickness is proportional to the removal rate from the liquid. Moreover, the presence of surface‐active compounds may bring about the thickening of the formed film (Lumanlan et al. 2020). These compounds are formed during frying as a result of chemical reactions such as polymerization, hydrolysis, and oxidation (Liberty et al. 2019). They can impact heat transfer at the oil‐food interface, thus giving rise to the contact area between the oil and the food and reducing surface tension; consequently, they elevate oil absorption (Xie et al. 2022; Zhang et al. 2020).

### 4.2 Mechanism of STO Absorption (Water Replacement)

Oil absorption into the pores of fried food, created due to water evaporation, is mainly explained by this mechanism, because there is a positive correlation between moisture loss and oil absorption during frying (Bouchon et al. 2003; Pedreschi et al. 2008). For example, rapid and continuous evaporation of water during deep‐fat frying led to structural changes in potato chips such as surface dehydration and development of a porous structure. The crust of French fries is approximately six times more porous than the core, indicating its greater oil absorption capacity (Lumanlan et al. 2020; Xie et al. 2022). During deep‐fat frying, the water present in the French fries evaporates, and a positive pressure gradient is created from the interior toward the exterior. The vapor produced during the process escapes from the cracks and open channels present in the membranes and the cellular structure, and an empty space is created for the oil penetration. As a result, the oil flows toward the large pores (Arslan et al. 2018) and increases the TOC of the product (Bouchon et al. 2003).

### 4.3 Mechanism of SO and PSO Absorption (Capillary Pressure)

Scientists introduced a physical relationship between oil absorption and porosity, expressing that the mechanism of oil absorption may be caused by capillary forces. Indeed, when fluid movement (like oil absorption) occurs in micro‐channels (like crust pores), surface phenomena such as viscosity and capillary forces highly assume importance (Lumanlan et al. 2020; Ziaiifar 2008). The pores present in food are of three types: (1) interconnected pores: accessible from any direction; (2) non‐connected pores: accessible from one direction; and (3) isolated pores: inaccessible (Lumanlan et al. 2020). Capillarity is the ability of narrow channels to draw a liquid upwards, which occurs when the intermolecular adhesion forces between the liquid and a solid are stronger than those in the liquid. This results in concavity where the liquid is in contact with a vertical surface (Lumanlan et al. 2020). Some researchers explained the capillary effect (according to the Equation (1)) by stating that the internal pressure of the food is higher than the atmospheric pressure during frying, owing to the resistance of the capillary tubes in the crust to the escape of water vapor (Patsioura et al. 2016). This phenomenon is the main factor in restraining the penetration of oil into the crust during deep‐fat frying (Patsioura et al. 2016). Capillary pressure (ΔPC), is a function of the contact angle (θC) and diverging angle (ϕ), as shown in Equation (1).
(1)

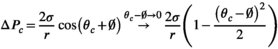

where *r* is the pore radius at the contact line. A tube is obtained when *ϕ* = 0. Considering a tube commensurate with the size of the parenchyma cell of potato, the average capillary pressure is 500 Pa, and oil absorption occurs when *P* < ΔPC. The Pearson correlation between the TOC and pore size of fried samples has revealed that pore size influences oil absorption (Zhang et al. 2020). Smaller pores bring about higher capillary pressures and thus higher TOC (Patsioura et al. 2016). Furthermore, a smaller contact angle between the oil and the product surface leads to greater adhesion forces and increased oil absorption.

### 4.4 Mechanism of PSO Absorption During Cooling (Vapor Condensation and Vacuum Effect)

The exact mechanisms of oil absorption during cooling have been partially known. Many studies have revealed that fried food absorbs the most oil after being removed from the fryer (Cortés et al. 2016; Patsioura et al. 2016). Even during frying, oil is not absorbed as much as during cooling, and it has been realized that the most oil absorption is associated with this stage (Yang et al. 2019). During frying, if the product temperature exceeds water boiling point, condensation of water vapor and vacuum effect will be observed in it during cooling. The solid matrix of food is an obstacle to the growth of water vapor bubbles, which results in a pressure gradient in the food. During frying, when the product still has a high free water content which is prone to evaporation, the escape of water and its resulting excess pressure is a barrier to oil absorption.

In contrast, when the product is removed from the fryer, its core temperature declines, the water vapor condenses, and the pressure inside the product lowers considerably. The significant difference between the internal and external pressures of the product produces the “vacuum effect” causing the SO to penetrate into the product (Bouchon et al. 2003; Cortés et al. 2016). On the outer surface of the food, oil absorption occurs when drainage and adhesion are balanced after removing the food from the fryer. The surface properties of the crust created during deep‐fat frying substantially aid in oil absorption and distribution. It is essential to note that oil absorption during cooling takes place only in the product crust (Arslan et al. 2018; Cortés et al. 2016; Dehghannya and Ngadi 2021; Rani et al. 2023).

In conclusion, removing the SO from the product when the temperature is still high has been suggested as an effective way of reducing the TO of the final product (Rahimi et al. 2017). Researchers have cited that when fried potato chips are taken out of the fryer, they start to cool, bringing about a decrease in the internal pressure and the condensation of the water vapor; hence, because of the vacuum effect, the SO is drawn into the pores formed by the water escape (Bouchon et al. 2003; Cortés et al. 2016). If it is assumed that pores behave like capillary tubes, the pressure flowing inward by the capillary behavior can be calculated using the Equation (2) (Eichenlaub and Koh 2015):

(2) $$P_{a}-P_{b}=\frac{2\;\sigma \;\cos\;\theta }{r}$$

where *θ* denotes the contact angle between the oil and the product, *r* stands for the pore radius, and *σ* represents the oil surface tension (Eichenlaub and Koh 2015). This factor (vapor condensation and vacuum effect) can be the most important force affecting oil absorption in a porous medium (Bouchon et al. 2003; Cortés et al. 2016). In order to decrease oil absorption during cooling, various strategies have been introduced, including application of alternative frying technologies such as vacuum, hot‐air, and pressure frying (Liberty et al. 2019).

## 5 Effect of Different Frying Methods on the Absorption Dynamics of Oil Fractions

Frying method and processing conditions, including frying temperature, frying time, and pressure, are among the major factors influencing oil absorption and the overall quality of fried foods by affecting moisture removal, crust formation, pore structure development, and capillary‐driven oil uptake during cooling (Lalam et al. 2013; Liu, Tian, Zhang, and Fan 2021). Consequently, optimizing these processing parameters can substantially reduce oil uptake while maintaining desirable texture and sensory quality (Liberty et al. 2019; Lumanlan et al. 2020; Zhang and Fan 2021). For this reason, various solutions have so far been offered. The efficiencies of these methods can be considerably different in diminishing oil absorption and retaining the textural and organoleptic properties of fried foods, as each of these methods has a unique effect on the oil absorption behavior which will be further discussed. Although all frying technologies aim to reduce oil uptake, the available evidence indicates that they do not achieve this through a common mechanism (Ghaitaranpour, Koocheki, et al. 2018; Liu, Tian, Duan, et al. 2021). Rather, each technology modifies one or more stages of oil absorption, including moisture removal, crust development, surface oil retention, pressure‐driven flow, or cooling‐induced capillary suction. Moreover, direct comparison among published studies remains difficult because frying temperature, product geometry, food composition, and oil type differ substantially across experiments. Consequently, the apparent superiority of one frying method over another should be interpreted cautiously, and mechanistic comparisons are generally more meaningful than simple comparisons of total oil content.

### 5.1 Deep‐Fat Frying

Deep‐fat frying is one of the oldest cooking methods, which has probably originated from the Mediterranean region. During this process, heat is transferred from the oil to the food surface through convection and then to its core through conduction, and moisture escapes through the food cracks and the pores created by the vapor pressure in the food. As the moisture is lost, the food surface temperature is elevated and approaches the temperature of the frying oil, which further leads to the formation of the crust, and the oil may also replace some of the escaped water (Ghaitaranpour et al. 2024; Ghaitaranpour, Koocheki, et al. 2018). As a heat transfer agent, oil develops a crispy texture in products such as potato chips, French fries, and fried chicken, in addition to cooking the food. The resulting product is usually brown to golden in color. The rate and efficiency of the frying process depend on the temperature and overall quality of the oil in terms of triglyceride degradation and changes in its thermal and physical properties such as color and viscosity (Thongcharoenpipat and Yamsaengsung 2021; Yang et al. 2019). High oil temperatures cause rapid heat transfer, quick browning, and short frying durations (Liu, Tian, Zhang, and Fan 2021).

Frying temperature is an important parameter controlled during frying to ensure the proper quality of fried foods. A non‐optimal frying temperature causes oil degradation, increased surfactant formation, product darkening, and more oil absorption. At the beginning of the frying process, moisture content is remarkably decreased, followed by a gradual reduction in the amount of evaporation (Asokapandian et al. 2020; Liu, Tian, Zhang, and Fan 2021). Opposing findings have been reported regarding the effects of frying time and temperature on oil absorption in deep‐fat‐fried foods. However, these apparent discrepancies do not necessarily reflect conflicting mechanisms; rather, they largely arise from differences in experimental design. Many studies evaluated only the final total oil content without distinguishing between surface oil, penetrated surface oil, and structural oil, while others did not account for the different stages of the frying process. Consequently, studies measuring only TOC often reached conclusions that differed from those separately quantifying individual oil fractions. Therefore, distinguishing oil fractions and considering the successive stages of frying are essential for accurately interpreting the influence of processing conditions on oil uptake and for reconciling seemingly contradictory findings in the literature.

**FIGURE 4:**
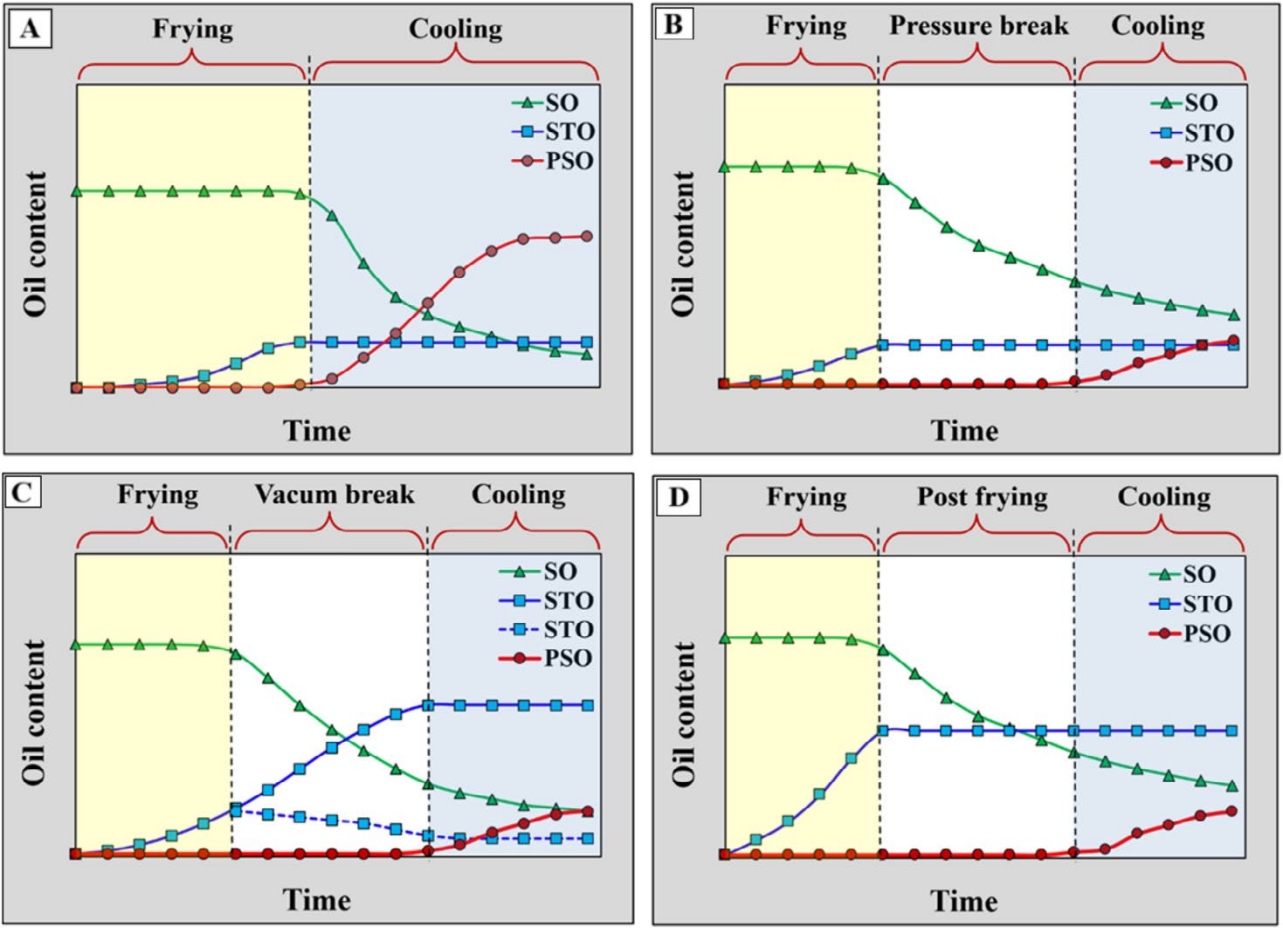
Schematic diagram of the changing pattern in amount of different oil categories (SO: Surface oil, PSO: Penetrated surface oil, STO: Structural oil) during frying: (A) deep‐fat frying, (B) pressure frying, (C) vacuum frying, (D) microwave frying.

The TOC of a deep‐fat‐fried product is absorbed in the two stages of frying and cooling, and the absorption extents of STO, SO and PSO are different in each stage. During deep‐fat frying, oil absorption begins immediately after the product surface is slightly dried. STO constitutes a low percentage of the TOC of the fried product in all the frying stages. This can be due to the rapid escape of water and the successive formation of bubbles on the product surface, which inhibits the oil absorption into the porous structure (Figure 4A). During frying, most of the oil remains on the surface of the product (SO) (Figure 4A), which is mainly absorbed after the product is removed from the fryer. During frying, intense dehydration and chemical reactions result in a rise in the crust thickness and porosity. This allows the oil to penetrate into the food after being removed from the fryer. During cooling, after the removal of the product from the frying medium, temperature drop has a substantial effect on oil absorption. The product temperature starts to decrease quickly after it leaves the fryer.

At temperatures below 100°C, water vapor condenses, and the internal pressure lowers; as a result, a vacuum (negative pressure) is created, which causes the SO (which was collected on the product surface during frying) to be sucked rapidly. As illustrated in Figure 4A, PSO constitutes the largest fraction of TOC in this stage (Ghaitaranpour et al. 2021; Lumanlan et al. 2020). As mentioned earlier, frying time and temperature affect the amount of heat transferred to the product. Therefore, they are closely related such that if the frying time exceeds an optimal limit at a constant temperature, oil absorption rises. High levels of oil absorption have also been reported at low frying temperatures because the product must remain in the fryer for a longer period to reach the desired degree of cooking, thereby increasing cumulative moisture loss and providing more opportunity for oil uptake (Asokapandian et al. 2020).

### 5.2 Pressure Frying

Although the use of pressure frying is limited in large‐scale industrial processing, it is widely employed in restaurants, delicatessens, supermarkets, schools, and other commercial food service operations (Pankaj and Keener 2017). More recently, researchers have investigated combining pressure frying with different coating formulations to further reduce oil absorption (Zhang et al. 2020). Pressure frying is conducted in a sealed, pressurized system, where pressure is generated by water vapor released from the food and, in some systems, supplemented with nitrogen gas. This technology is primarily applied to meat, fish, and poultry products because it accelerates heat transfer, shortens frying time, and improves moisture retention, resulting in products with superior texture (Innawong et al. 2006; Su, Gao, Tang, et al. 2022). Although pressure frying consistently reduces oil absorption compared with conventional deep‐fat frying, the underlying mechanisms remain incompletely understood. Most studies attribute this reduction to the combined effects of enhanced heat transfer, reduced frying time, and improved moisture retention. However, these factors alone may not fully explain the observed decrease in total oil content, because pressure frying uniquely includes a pressure‐release stage that is absent in conventional frying. In order to better understand the mechanism of oil absorption in pressure frying, this process has been divided into the three stages of frying, pressure breaking, and cooling (Figure 4B).

As can be seen in Figure 4B, in the frying stage, as the product surface is dried as a result of water evaporation, oil absorption begins, and the product STO starts to increase with a gentle slope. At this stage, most of the oil remains on the product surface (SO). This type of oil is absorbed during cooling. It seems that shorter frying time and higher moisture retention in the food product are not the only reasons why oil absorption is lower in pressure frying compared to deep‐fat frying. Since there is also a pressure breaking stage in pressure frying, SO and STO are reduced, likely because the pressure release causes the product's contents (vapor and liquids) to escape, which prevents oil from entering the product and may even cause some absorbed oil to escape during frying. As shown in Figure 4B, the last stage of oil absorption occurs during cooling which is started after the product is removed from the fryer. Since the most oil absorption takes place in this stage, a fraction of SO is absorbed in this stage (PSO). The extents of moisture retention and oil absorption reduction are ascribed to the amount of the pressure applied to the fried product (Pankaj and Keener 2017). Researchers have also demonstrated that the TOC decreased significantly with a rise in the pressure, so that high pressures resulted in lower TOC than low pressures (Pankaj and Keener 2017; Zhang et al. 2014). Taken together, current evidence suggests that pressure frying reduces oil absorption through multiple interacting mechanisms rather than a single dominant effect. Nevertheless, most published studies report only the final oil content without distinguishing individual oil fractions, making it difficult to determine the relative contribution of reduced frying time, improved moisture retention, and pressure release. Future work employing real‐time oil tracking and fraction‐specific analysis would substantially improve mechanistic understanding.

### 5.3 Vacuum Frying

As mentioned before, in recent years, consumers have been seeking fried products with lower fat content that maintain the principal characteristics of the food (Pan et al. 2015). Vacuum frying technology has been described as an alternative to deep‐fat frying to improve the quality of fried food (Rosida et al. 2018). It is a process conducted at pressures lower than the atmospheric pressure and has been used for a variety of products such as apple, banana, carrot, mushroom, potato, and sweet potato (Motamedzadegan et al. 2016; Soto et al. 2021). This process encompasses several steps including heating the oil, placing the product in the fryer chamber and closing the lid, decreasing the pressure, immersing the product in the oil, frying, and removing the oil (through centrifugation) during processing. The equipment utilized for this process includes a fryer chamber, a vacuum pump (water vacuum pump or oil vacuum pump), an oil removal device, and a condenser (Banerjee and Sahu 2017; Diamante et al. 2015; Pandey et al. 2020; Sosa‐Morales et al. 2022). Functionally, this equipment is applied in batch and continuous forms (Sosa‐Morales et al. 2022), and the former is expensive (Pankaj and Keener 2017). In the batch type, the oil is heated using a gas, electric, or steam burner installed under the fryer chamber. The vacuum pump is also employed with high intensity to create the vacuum before frying. The pieces of the food to be fried are placed inside the fryer basket which can be dipped in hot oil to start the frying process during which moisture evaporates from the food surface and moves toward the condenser through vacuum, where it is condensed and changed into liquid using the cooling coils and collected in a separate chamber (Diamante et al. 2015; Pandey et al. 2020; Sosa‐Morales et al. 2022). The frying operation is performed in a sealed chamber under pressures much lower than the atmospheric pressure. Since the temperature of oil is low in this process, and the boiling point of water lowers, leading to more decrease in the oil temperature during the process (Ayustaningwarno et al. 2020; Correa et al. 2017; Negara et al. 2021; Pandey et al. 2020; Rosida et al. 2018; Zhang et al. 2020).

Low frying temperatures and the absence of oxygen are the reasons behind most of the benefits of vacuum‐fried products (Bedoya et al. 2018; Chen et al. 2021; Correa et al. 2017; Ghaitaranpour, Mohebbi, and Koocheki 2018; Maity et al. 2015; Pan et al. 2015; Thongcharoenpipat and Yamsaengsung 2021), one of which is the reduced TOC. The mechanism of oil absorption in vacuum frying can be divided into three stages including frying, vacuum breaking, and cooling (Figure 4C).

#### 5.3.1 Oil Absorption During Frying

At the beginning of this process, the percentage of oil absorption is lower than in conventional frying. This could be due to the lower frying temperature and decreased texture destruction (Ayustaningwarno et al. 2020; Correa et al. 2017; Motamedzadegan et al. 2016; Soto et al. 2021; Wexler et al. 2016). Moreover, the water present in the food evaporates and is partially replaced with the oil. After this stage, the food is removed from the oil, and vacuum breaking begins, which also impacts oil absorption.

#### 5.3.2 Oil Absorption During Vacuum Breaking

Considerable disagreement exists regarding the role of vacuum breaking in oil absorption. While several studies concluded that vacuum frying reduces overall oil uptake compared with atmospheric frying, others observed increased oil penetration during pressure equilibration. These apparently conflicting findings are likely attributable to differences in vacuum‐breaking rate, product microstructure, moisture content, and the application of centrifugal deoiling. Therefore, vacuum breaking should not be regarded as either beneficial or detrimental in isolation; instead, its effect depends strongly on processing conditions.

Generally speaking, oil absorption during vacuum frying is primarily driven by the vacuum breaking process. When the vacuum is released, the surrounding pressure increases (Figure 4C), causing water vapor to condense and oil (Figure 4C, solid line—STO) to be drawn into the product until the pressure stabilizes at atmospheric levels. Consequently, oil absorption is influenced by both the surface oil (SO) and the moisture content of the product (Mir‐Bel et al. 2009; Soto‐Jover et al. 2016). However, since there is a linear relationship between oil absorption and the inverse of the vacuum breaking rate, vacuum breaking does not always raise the oil absorption (Figure 4C, dashed line—STO). In other words, rapid vacuum breaking can significantly reduce the product's total oil content (TOC), primarily due to the compression of the product caused by the sudden pressure change (Figure 4C). This compression reduces the product's volume, leading to less internal space available for oil absorption.

#### 5.3.3 Oil Absorption During Cooling

Changes in the TOC of vacuum‐fried products continue even during cooling; the stage in which the product is removed from the fryer. Absorption of SO is higher in vacuum frying than in the conventional one. Therefore, a deoiling system (centrifugation) is employed immediately after frying and before vacuum breaking or cooling. It is also possible to decrease the absorption of SO by further reducing the pressure in the fryer as the product is being removed from the fryer chamber (AlMohaimeed 2017; Dehghannya and Ngadi 2021; Mir‐Bel et al. 2009; Soto‐Jover et al. 2016). As exhibited in Figure 4C, the absorption of SO in vacuum‐fried products continues during cooling, similar to deep‐fat‐fried ones, even though to a much lesser extent (Mir‐Bel et al. 2009; Soto‐Jover et al. 2016). The available evidence indicates that the success of vacuum frying depends less on operating under reduced pressure itself than on carefully controlling the transition back to atmospheric pressure.

### 5.4 Microwave Frying

Microwave processing is a relatively new heating method applied in a variety of sectors, especially in the food industry. Microwave heating has many advantages including fast heating, high temperature, short cooking time, and low operating cost. Furthermore, this technique can enhance the safety and quality of food products (Nguyen and Songsermpong 2022). Electromagnetic waves have been utilized since the 1940s. Food industry is the largest user of the microwave energy in processes such as drying, blanching, cooking, defrosting, pasteurizing, sterilizing, baking, heating, and fat melting (Kishimoto 2019; Michalak et al. 2020).

The frequencies of microwaves range from 300 MHz to 300 GHz, and the corresponding wavelengths vary between 1 mm and 1 m (Michalak et al. 2020; Nguyen and Songsermpong 2022; Verma et al. 2020). Theoretically, the mechanism of microwave heating is based on ion polarization and the dipole rotation of charged groups. Microwave heating is a thermal process and an alternative heating method in the food processing industry. Microwave heating results from the ability of products to absorb the microwave energy and convert it into heat. Water is the main ingredient that causes foods to be heated by microwaves. The higher the moisture content of the food, the faster the heating rate. Except for the water molecules, the food polar particles are also affected by the strong electromagnetic field, resulting in the rotation of the polar molecules. The very high rotation speed causes the water molecules and polar particles of the food to collide with each other at a very high speed. This brings about friction between the molecules and heat production. In traditional heating processes, the energy is transferred from the product surface to its interior through conduction, which highly depends on the temperature gradient and thermal conductivity of the product. Compared with traditional heating methods, microwaves lead to the volumetric heating of the food and create a noteworthy vapor pressure gradient between its center and outer surface (Barutcu and Bekir 2017; Jumras et al. 2020; Michalak et al. 2020; Nguyen and Songsermpong 2022; Sansano et al. 2018; Verma et al. 2020; Wang et al. 2021).

Compared with pressure and vacuum frying, the evidence for microwave frying is considerably less consistent. Some studies reported lower oil uptake at high microwave power, whereas others observed increased oil absorption caused by structural damage and rapid moisture escape. These conflicting observations suggest that microwave power alone cannot predict oil absorption behavior; instead, the interaction among microwave intensity, heating uniformity, and food microstructure appears to be the determining factor. Compared to deep‐fat frying, it has been realized that the temperature of microwave frying linearly increases with time until it reaches the boiling point of water. After that, temperature is elevated in the range of 100°C–103°C for a short period of time and then fluctuates around 100°C for the rest of the frying stage (Parikh and Takhar 2016). During deep‐fat frying, the frying temperature later reaches the water boiling point, compared to microwave frying. Microwave heating leads to rapid evaporation of water, followed by the creation of high pressure, particularly in the central parts of the product, which results in the outflow of the water vapor from the internal parts, thereby accelerating the heat transfer. The rapid moisture loss in this method can cause increased oil absorption, compared to deep‐fat frying (Naik et al. 2017; Parikh and Takhar 2016).

It seems that volumetric heating, the reason behind microwave frying, creates structural channels in the food matrix, which causes oil absorption in microwave‐fried products. As pointed out in numerous studies, although it appears that oil absorption is sometimes lower in microwave frying than in the conventional one, it is not significant. Some studies have regarded the high microwave power as the reason for the decrease in oil absorption, which brings about a rise in the rate of water escape from the product and consequently restrains oil absorption (Aydınkaptan and Mazı 2017; Luna 2019; Naik et al. 2017; Noor Hidayati et al. 2021; Sansano et al. 2018). It has also been stated that when the magnetron stops, there is no input electromagnetic energy to the frying chamber. Therefore, water less escapes toward the food surface, and as a result, oil absorption increases. This can also explain why more oil is absorbed in microwave frying (Sansano et al. 2018). Oil absorption after microwave frying has also been studied. It has been claimed that the TOC of the samples removed from the microwave oven and cooled at room temperature remained unchanged during the post‐frying period (Figure 4D) (Zhou et al. 2022). Nevertheless, the TOC of the samples diminished when they were stored in the microwave oven where the air temperature was high, probably owing to the high air temperature at which the samples were kept. The decrease in the TOC can be ascribed to the reduced oil viscosity at high temperatures. Less viscous SO can be separated from the product surface more easily, resulting in less oil absorption. Pore pressure can be another effective factor, which can likely be increased in the food crust by focusing the microwaves on it until the oil is removed from the product. In practice, after the frying, the food baskets are shaken to partly separate the SO and lower the TOC of the fried product (Zhou et al. 2022).

Microwave frying has advantages such as retention of color and flavor, reducing acrylamide, and preserving antioxidants (Hu et al. 2021; Kishimoto 2019; Nguyen and Songsermpong 2022; Sansano et al. 2018; Taher Ahoussein et al. 2018). At the same time, application of this method alone is not still common, because the excessive heating rate of microwaves destructs the internal structure of the sample, in addition to some other factors that are not easy to control. Consequently, when microwave technology is going to be used for food processing, it is often applied in combination with other technologies such as hot air, vacuum, and drying (Hu et al. 2021).

### 5.5 Hot‐Air Frying

Hot‐air frying is a novel method of producing fried food with less TOC and the same appearance and taste as conventionally‐fried food (Fikry et al. 2021; Ghaitaranpour et al. 2021). In this method, oil is sprayed on the food which is surrounded by hot air instead of being immersed in the oil (Devi et al. 2021; Ghaitaranpour et al. 2021; Gouyo et al. 2021). In domestic devices built on this basis, hot air is in direct contact with the food and the oil droplets present on its surface. These devices are often designed such that heat is transferred uniformly and at a high rate between the hot air and the food (Ghaitaranpour et al. 2021; Gouyo et al. 2021; Tian et al. 2017; Wang et al. 2021; Zaghi et al. 2019). There is only one blower in some devices, whereas in some other types, heat may be transferred through radiation in addition to convection. In some cases, the design of the device frying chamber is such that the hot air flowrate is higher in it than in the conventional one, or in other words, the hot air is distributed more uniformly around the food, so that quality changes in its different parts will be minimized (Teruel et al. 2015). As mentioned earlier, hot air is the heat transfer medium in this method. Therefore, a much smaller amount of oil is in contact with the food during the process (Wang et al. 2021). In recent years, a great deal of attention has been paid to this method of frying in such a way that the domestic version of hot‐air fryer has been constructed (Ghaitaranpour et al. 2021).

The technology of hot‐air frying was first introduced in some European markets, and owing to its remarkable reception, it was offered throughout Europe. Over time, it expanded on the global scale and became a huge success worldwide. Cooking products with this technology keeps their taste pleasant and their TOC much lower, compared to deep‐fat frying (Zaghi et al. 2019).

Hot‐air frying has benefits such as reducing oil absorption (Negara et al. 2021), preserving fat‐soluble vitamins due to the absence of oil as the heat transfer medium, and the negligible effect of oil changes on the food quality during the frying. During this process, dehydration begins, and a crust is gradually formed on the product. Oil spraying on the product, which is carried out to develop its texture, color, and flavor similar to those of deep‐fat‐fried ones, can be done before or during the frying process. The amount of the oil consumed in this method, is significantly smaller than that in deep‐fat frying, thus producing fried products with very low TOC (Abd Rahman et al. 2017; Ghaitaranpour et al. 2021; Wang et al. 2021).

**FIGURE 5:**
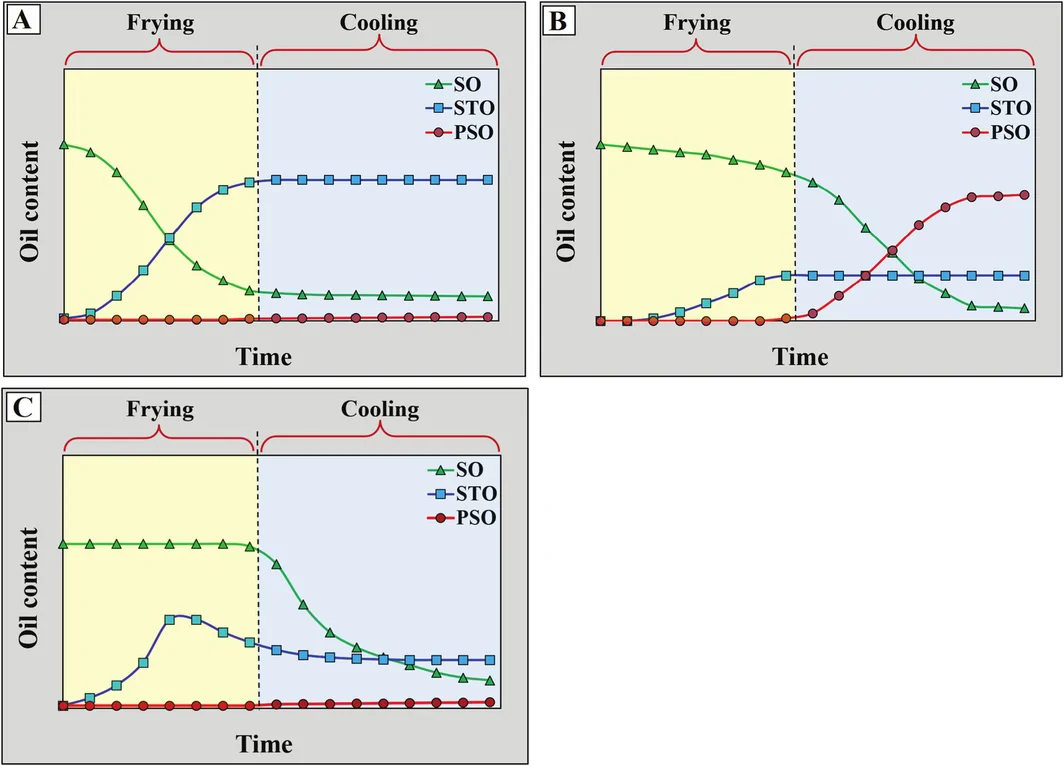
Schematic diagram of the changing pattern in amount of different oil categories (SO: Surface oil, PSO: Penetrated surface oil, STO: Structural oil) (A) hot air frying, (B) IR‐frying, (C) G‐frying.

In deep‐fat frying, the product is in contact with a large volume of oil, whereas in hot‐air frying only a small amount of oil, typically sprayed onto the product before frying, is available. Consequently, much less SO remains on the product after frying, leaving little oil available for capillary‐driven uptake during cooling. As a result, oil absorption during the cooling stage is negligible (Figure 5A), and the lower total oil content TOC of hot‐air‐fried products is primarily attributed to the limited availability of surface oil rather than changes in the cooling‐stage absorption mechanism itself (Ghaitaranpour et al. 2021). This distinguishes hot‐air frying from pressure and vacuum frying, where reductions in TOC are largely associated with modifications in pressure‐driven oil transport during or immediately after frying. Therefore, comparing frying methods solely on the basis of final TOC may obscure the fundamentally different mechanisms by which oil reduction is achieved. Researchers have revealed that in deep‐fat frying, oil absorption chiefly occurs during the cooling stage rather than the frying one. In the case of oil absorption during the cooling stage of hot‐air frying, it has been declared that it is ignored due to the absence of a significant amount of oil on the food surface. Hot‐air frying can be divided into the two stages of frying and cooling to better show the TOC changes in the food during this process (Figure 5A).

#### 5.5.1 Oil Absorption During Frying

The frying stage can be described in three principal phases as follows (Figure 5A): (1) Latent phase which is the shortest phase of oil absorption in hot‐air frying. The stage of no oil penetration is reached a short time after the beginning of the frying process. Hence, oil penetration into the crust is ignored, as the amount of the water evaporated from the crust is not still enough to start the oil penetration. The duration of the latent phase depends on several factors including the frying temperature with a rise of which, the duration of the latent phase declines. (2) Acceleration phase which is the most effective phase of oil penetration. Most of the crust moisture evaporates in this stage. As a result, the major part of the SO can penetrate into the crust quickly. (3) Oil absorption rate decline, where oil penetration continues with a very gentle slope. The effect of this phase on oil absorption is less pronounced than that of the acceleration one. The duration and effect of this phase on oil absorption increase as the fryer temperature is lowered (Figure 5A) (Ghaitaranpour et al. 2021).

#### 5.5.2 Oil Absorption After Frying

The cooling stage, during which the most oil is absorbed in deep‐fat frying, can be ignored in the hot‐air method, because no oil absorption takes place in this stage owing to the absence of a substantial amount of SO (Figure 5A).

### 5.6 Infrared Frying

Infrared is a part of the electromagnetic spectrum that lies between the visible region and microwaves. Its wavelength is from 0.5 to 100 μm (Aboud et al. 2019). Based on the wavelength, IR radiation can be divided into three regions: near IR (NIR) with wavelength from 0.75 to 1.4 μm, mid‐IR (MIR) with wavelength between 1.4 and 3 μm and far‐IR (FIR) with wavelength from 3 to 1000 μm (Aboud et al. 2019; Semwal and Meera 2021; Udomkun et al. 2019). IR‐Far can be used in food processing because most food components can absorb radiant energy at this wavelength (Udomkun et al. 2019). In general, IR radiation is electromagnetic radiation that is transmitted in the form of waves and when it hits the food surface (water molecules and ions) it can increase the surface temperature (Rahimi et al. 2018; Semwal and Meera 2021; Su, Gao, Chen, et al. 2022; Udomkun et al. 2019). The depth of IR penetration depends on the thickness of the product, water activity, product components and the infrared wave length. Infrared heating has numerous applications in different food processing operations such as drying, pasteurizing, baking, sterilizing and blanching (Devi et al. 2021; Rahimi et al. 2018). Recently, the possibility of using infrared frying has been considered as an alternative frying method due to its faster heating rate, heat distribution and more uniform moisture removal as a result of decreasing heating time and reducing product's oil content (Su, Gao, Tang, et al. 2022). In IR frying, thanks to faster heat transfer, the rate of moisture loss is higher in comparison with deep fat frying. The rate of moisture evaporation gradually decreases as frying progresses. Moisture evaporation at the exterior part of the product creates a specific crust layer. In starchy fruits, infrared frying leads to quicker crust formation with a harder and smoother surface, which minimizes oil adhesion on the surface and within the crust's internal structure in fried products (Su et al. 2025). As shown in Figure 5B, SO and STO decrease and increase respectively during IR‐frying. They start to change in different time segments because each oil fraction has its own special mechanism for being observed. SO has a direct relation with the surface morphology of the fried samples, while, STO influenced by the porous characteristics of samples. Changing in the morphology of the crust starts earlier than variation in products porosity. The initial rapid oil penetration period in IR‐frying (Figure 5B) follows by more or less constant stage (Su, Chen, et al. 2024; Su, Gao, Chen, et al. 2022; Udomkun et al. 2019). Although Lake of moisture to escape from the product reduces the oil absorption at the final stages of IR‐frying, the early‐stage crust formation in starchy fruits during frying can act as a physical barrier to moisture release, increasing the pressure gradient. Over time, this leads to the development of large pores and cracks in the crust, allowing more surface oil to penetrate the interior structure (PSO) during the cooling phase (Su et al. 2025). Although recent studies consistently report reduced oil uptake during infrared frying, the available evidence remains limited compared with conventional, pressure, or vacuum frying. Consequently, the proposed mechanisms should currently be regarded as promising rather than fully established, particularly because quantitative information regarding individual oil fractions is still scarce.

### 5.7 G‐Frying

As already mentioned, oil absorption is a superficial phenomenon which mainly occurs at the beginning of the frying process. The impact mechanism of the cooling stage provides the necessary driving force which causes a considerable amount of oil to be absorbed after the frying process. A layer of oil on the food surface, which is removed from the fryer along with the food, constitutes almost the largest percentage of the TOC of the final product (Kim and Moreira 2013; Odunlami et al. 2023; Sothornvit 2011). Various solutions have been suggested and used for removing SO, including using absorbent paper or washing fried products with petroleum ether or hexane, which are not desirable respectively because of insufficient control over the conditions such as time, temperature, and hand pressure and the risk of chemical residues in the products. According to the literature, shaking and centrifuging the product immediately after frying can be a good way of removing its SO. G‐frying refers to the application of centrifugal forces during or after the frying (Khalilian et al. 2021; Odunlami et al. 2023). In this method, the fryer has a centrifuge system consisting of a basket that rotates during frying. When the temperature of the frying oil reaches the desired value, the samples are placed inside a wire basket in the frying chamber and then immersed in the hot oil (Khalilian et al. 2021; Sothornvit 2011). Oil absorption in G‐frying has shown different behaviors in different products, which can be attributed to the changes in the crust and microstructure of the product during frying, where the structure of the product internal pores is damaged, causing the number of the open pores to decline. Additionally, the lack of crust formation in some fried products makes it possible for the oil to easily diffuse out of the fried samples and enter into the frying medium. At the same time, the TOC of the food generally rises at the beginning of the frying stage and then decreases (Figure 5C). Compared with conventional frying, G‐frying leads to an increase in heat transfer rate and accelerates the separation of the vapor bubbles from the product surface.

In other words, when the food is immersed in the oil parallel to the basket rotation direction, the centrifugal force removes the vapor bubbles from around the food and makes the hot oil come into closer contact with the food; consequently, the heat and mass transfer rates are enhanced. This results in more moisture loss in the fried food compared to deep‐fat‐fried products (Khalilian et al. 2021; Kim and Moreira 2013). After frying, G‐frying applies centrifugal force to remove surface oil retained on the product, thereby reducing the final TOC (Figure 5C). The deoiling efficiency depends on the orientation and structural characteristics of the product, with vertically positioned samples generally exhibiting higher oil removal rates (Sothornvit 2011). However, centrifugation speed and cooling time have shown limited effects on the final TOC under certain conditions, suggesting that the effectiveness of post‐frying deoiling is not solely determined by processing parameters but is also influenced by product geometry and mechanical stability (Khalilian et al. 2021; Sothornvit 2011). Among the reviewed frying technologies, G‐frying is unique because it primarily reduces oil content by actively removing surface oil after frying rather than by altering oil penetration mechanisms during frying. Therefore, minimizing oil adhesion at the product surface may represent an effective strategy for reducing oil uptake, particularly for products in which surface oil constitutes a major fraction of TOC. Nevertheless, further studies are required to determine how product structure and mechanical properties influence the efficiency and scalability of centrifugal deoiling.

## 6 Conclusion and Future Perspectives

This review provides a comprehensive comparison of oil absorption behavior across conventional and emerging frying technologies by examining the migration of individual oil fractions, namely surface oil (SO), penetrated surface oil (PSO), and structural oil (STO), rather than considering total oil content (TOC) alone. The comparison demonstrates that oil uptake is governed by different mechanisms depending on the frying technology. While deep‐fat frying is characterized by substantial oil absorption during the cooling stage, hot‐air frying exhibits negligible post‐frying oil uptake, G‐frying is associated with a marked reduction in SO during cooling with relatively stable PSO and STO, and pressure and vacuum frying are strongly influenced by pressure changes following processing. These findings indicate that the effectiveness of emerging frying technologies depends not only on reducing total oil uptake but also on altering the migration pathways of specific oil fractions. This fraction‐based perspective provides a more mechanistic understanding of oil absorption and may support the development of healthier fried products without compromising desirable sensory characteristics.

The findings of this review have practical implications for both researchers and the food industry by identifying technology‐specific mechanisms that can be exploited to optimize frying conditions, improve product quality, and reduce oil content. Nevertheless, several limitations should be acknowledged. The available literature remains heterogeneous with respect to food materials, frying conditions, oil types, and analytical methods used to quantify oil fractions, making direct comparisons among studies difficult. Furthermore, fraction‐specific data are limited for several emerging frying technologies, particularly G‐frying, restricting comprehensive mechanistic evaluation. Future research should therefore focus on standardized methodologies for oil fraction analysis, quantitative investigation of fraction‐specific migration mechanisms, and validation of these technologies under industrial‐scale processing conditions. Such studies will facilitate the design of next‐generation frying technologies capable of simultaneously improving nutritional quality, processing efficiency, and consumer acceptance.

## Author Contributions

**Arash Ghaitaranpour:** writing – review and editing, writing – original draft, supervision, methodology, project administration, conceptualization, investigation. **Mohsen Esmaiili:** writing – review and editing, methodology. **Zoleykha Fahimi vajargahi:** writing – original draft. **Michael O. Ngadi:** writing – review and editing.

## Funding

The authors have nothing to report.

## Ethics Statement

The authors have nothing to report.

## Conflicts of Interest

The authors declare no conflicts of interest.

## Data Availability

No data was used for the research described in the article.
